# Randomized, Single Blind, Controlled Trial to Evaluate the Prime-Boost Strategy for Pneumococcal Vaccination in Renal Transplant Recipients

**DOI:** 10.1371/journal.pone.0046133

**Published:** 2012-09-28

**Authors:** Selma Tobudic, Veronika Plunger, Gere Sunder-Plassmann, Markus Riegersperger, Heinz Burgmann

**Affiliations:** 1 Department of Internal Medicine I, Division of Infectious Diseases and Tropical Medicine, Medical University of Vienna, Vienna, Austria; 2 Department of Internal Medicine III, Division of Nephrology and Dialysis, Medical University of Vienna, Vienna, Austria; Health Protection Agency, United Kingdom

## Abstract

Renal transplant recipients are at increased risk of developing invasive pneumococcal diseases but may have poor response to the 23-valent pneumococcal polysaccharide vaccine (PPV). It may be possible to enhance immunogenicity by priming with 7-valent pneumococcal conjugate vaccine (7vPnC) and boosting with PPV 1 year later. In a randomized single-blind, controlled study, adult recipients of renal transplants received either 7nPVC or PPV followed by PPV 1 year later. The vaccine response was defined as 2-fold increase in antibody concentration from baseline and an absolute post-vaccination values ≥1 µg/ml. The primary endpoint was vaccine response of the primed group (7vPnC/PPV) compared with single PPV vaccination. Antibody concentrations for 10 serotypes were measured at baseline, 8 weeks after first vaccination, before second vaccination, and 8 weeks after second vaccination. Of 320 screened patients, 80 patients were randomized and 62 completed the study. Revaccination with PPV achieved no significant increase of immune response in the 7vPnC/PPV group compared with the single PPV recipients A response to at least 1 serotype was seen in 77.1% of patients who received 7vPnC and 93.1% of patients who received PPV (*P* = 0.046). After second vaccination response to at least 1 serotype was seen in 87.5% patients of 7vPnC/PPV group and 87.1% patients of PPV group (non significant p). The median number of serotypes eliciting a response was 3.5 (95% CI 2.5–4.5) in the 7vPnC/PPV group versus 5 (95% CI 3.9–6.1) in the PPV group (non-significant p). Immunogenicity of pneumococcal vaccination was not enhanced by the prime–boost strategy compared with vaccination with PPV alone. Administration of a single dose of PPV should continue to be the standard of care for adult recipients of renal transplants.

**Trial Registration:**

EudraCT 2007-004590-25.

## Introduction

*Streptococcus pneumoniae*, a common pathogen colonizing the upper respiratory tract, causes substantial morbidity and mortality from non invasive diseases such as otitis media and sinusitis to invasive diseases, including pneumonia, septicaemia and meningitis. The risk of invasive pneumococcal diseases among renal transplant recipients is more than 60 times higher than in the general population [1]–[3]. Pneumococcal polysaccharide vaccine (PPV), a 23-valent vaccine derived from the capsular polysaccharide of *S. pneumonia*e, is widely used and is recommended by WHO for all individuals exceeding the 60^th^ and the 65^th^ year of life, and for those with underlying chronic illness and pre- and post-splenectomy. The PPV includes 23 serotypes and thus provides the broadest coverage of the pneumococcal vaccines available. A single dose of this vaccine is therefore recommended for all patients after they undergo solid-organ transplantation, with a single additional dose of vaccine given 3–5 years later [4]. However, the use of immunosuppressive medication such as cyclosporine, prednisone, and mycophenolate mofetil may impair patients’ response to vaccination [5]. Recently published data have shown a suboptimal vaccine response to PPV in heart transplant patients [6]. Conjugate pneumococcal vaccines have been developed in order to enhance the immunogenicity of the polysaccharide vaccine. The 7-valent conjugate vaccine (7vPnC), which is licensed for vaccination of children, elicits a T cell-dependent immune response and has shown immunogenicity in a healthy adult population [7] and demonstrated immunogenicity similar to that for PPV in a randomized trial in adult recipients of kidney and liver transplants [8], [9]. The T cell-dependent response is more effective in producing memory B cells that may be boosted by subsequent polysaccharide antigen vaccination to produce a large amount of antibody. This prime–boost strategy has been shown beneficial in cohorts of patients with hereditary spherocytosis [10] and HIV infection [11], and several experts have suggested that such a strategy may improve vaccine immunogenicity in patients with solid-organ transplants [12]. However, other findings have shown that the immunogenicity of pneumococcal vaccine in adult liver-transplant patients is not enhanced by a prime–boost strategy when compared with vaccination with PPV alone [9].

We conducted a prospective randomized, single-blind, controlled trial in adult recipients of renal transplants to evaluate the immunogenicity of a prime–boost vaccination strategy using 7vPnC followed by PPV 12 months later in comparison with administration of standard single-dose PPV.

## Materials and Methods

### Patient Population

Adult renal-transplant recipients at the general hospital of Vienna who had undergone renal transplantation at least 6 months earlier were recruited to the study during the period November 2008 to October 2009. Exclusion criteria were previous splenectomy, pneumococcal vaccination within the preceding 5 years, ongoing treatment for an episode of allograft rejection, any acute febrile illness within 2 weeks prior to involvement, and use of intravenous immunoglobulin within the preceding 6 months. Randomization was computer generated with allocation concealed using opaque, sequentially numbered, sealed envelopes. To prevent subversion of the allocation sequence the name and date of birth of each participant was written on the appropriate envelope. Participants were randomized to receive either 7vPnC (0.5 ml, Prevenar®; Wyeth Q2 Pharma GmbH) or PPV (0.5 ml, Pneumo 23®, Merieux) for primary vaccination [4]. One year later, all patients received PPV vaccination.

All the patients were blinded to their group assignment. Serum specimens were collected at baseline, 8 weeks after the first dose of vaccine, before the second vaccination, and 8 weeks after the second dose of vaccine. All patients completed a 7-day diary after each dose to record systemic symptoms, oral temperature, and the diameter of injection site redness or swelling.

### Ethics Statement

The protocol for this trial and supporting CONSORT checklist are available as supporting information, see Checklist S1 and Protocol S1. Written informed consent was obtained from all patients and the study was approved by the ethics board of the institution (EudrCT: 2007-004590-25).

### Laboratory Methods

Quantitative pneumococcal antibody titers were determined for each of the 7 serotypes contained in both vaccines (4, 6B, 9V, 14, 18C, 19V, 23F) and for an additional 3 serotypes included only in PPV (1, 5, 7F) using an in-house isotype-specific reference ELISA according to the WHO protocol at http://www.vaccine.uab.edu/ELISA%20Protocol.pdf [13]. Briefly, 96-well microtiter plates Q4 (Greiner, Germany) were coated with previously determined optimal antigen concentrations (serotype 14∶1 mg/mL; serotypes1, 4, 5, 7F, 9V, 18C: 2 mg/mL; serotypes 6B, 19F, 23F: 5 mg/mL) and incubated at 37°C for 5 h in a humidified chamber. Coated plates were stored at 4°C and used within 3 weeks. Standard reference serum (89-SF), kindly provided by Carl E. Frasch (Center for Biologics, Rockville, MD, USA), was absorbed with 5 mg/mL of highly purified cell-wall polysaccharide (C-Ps) (Statens Serum Institute, Copenhagen, Denmark). Quality-control sera and test specimens were preincubated with C-Ps at 5 mg/mL and 22F capsular polysaccharide at 10 mg/mL final concentrations to remove cross-reactive antibodies. The coated plates were washed 5 times with 1×Tris-buffered saline containing 0.1% Brij 35 (pH 7.2), and then serially diluted reference serum, control sera, and test sera were added to the plates in duplicate wells and incubated for 2 h at room temperature. Plates were washed again as described above and 50 µL of 1∶5000 goat anti-human IgG-alkaline phosphatase conjugate (Sigma) Q5 was added to each well. After incubation for 2 h at room temperature, followed by a final washing step, the plates were developed with 100 µL of nitrophenyl phosphate substrate (Sigma) 1 mg/mL for 30 min at room temperature. The enzyme reaction was stopped with 50 µL of 3M NaOH and the absorbance measured at an optical density of 405 nm in an ELISA reader. Anti-pneumococcal IgG antibody concentrations were calculated with a weighted log-logit computer program. The interassay coefficient of variation was about 20%.

### Endpoint Definitions and Statistical Analysis

The primary endpoint was vaccine response of the primed group (7vPnC/PPV) compared with single PPV vaccination. Although the primary endpoint was the difference in antibody concentration as a continuous variable between study groups, we based our calculation of sample size on the more conservative but clinically more useful binary variable vaccine response. The response measured by ELISA was defined as a ≥ 2-fold increase in antibody concentration from baseline and an absolute post-vaccination value of at least 1 µg/ml. Although a value of 0.35 µg/ml has been defined as a correlate of protection for invasive disease in infant recipients of pneumococcal conjugate vaccine [14], the convenient cut-off value of 1.0 µg/ml was chosen because the true correlate of protection for adults is not known. The utility of this higher cutoff value has not been assessed in clinical trials [15].

The study was powered to detect an improvement in vaccine response with the 7vPnC/PPV vaccine schedule in any of the 7 serotypes compared with single PPV vaccination and was set to yield a power of 80% at a two-sided alpha level of 5% in order to detect a 35% improvement in the response rate for a given serotype. The response rate in the control group was expected to be about 45%. Thus the calculated simple size was 35 in each group. We added 10% to adjust for potential loss-to-follow-up.

Secondary endpoints were vaccine response after the primed 7vPnC/PPV regime compared with sequential PPV/PPV vaccination, decline of antibody response/vaccine response 1 year after 7vPnC or PPV vaccination, and tolerability of vaccine regimes.

The data were analyzed using SPSS 17.0 software. The two-tailed, unpaired Student’s test was used to compare geometric mean antibody concentrations (GMCs) for each pneumococcal serotype in the two groups after log transformation of the raw data, given that our distributional assumptions held. For hypothesis testing we used Fisher’s exact test.

Titers at 1 year were compared with those obtained 8 weeks after initial vaccination (7vPnC, PPV) for each serotype using the Wilcoxon signed rank sum test to investigate the influence of time on durability of vaccine response. The fold increase in ELISA concentrations were calculated by dividing the post-vaccination concentration by the pre-vaccination concentration. Differences in serotype responses between the 7vPnC/PPV and PPV/PPV groups were compared using the Mann-Whitney U test. To adjust the p-values for multiple serotypes Bonferroni correction was used. Logistic regression was used to explore available factors that might be associated with serotype response at indicated time points. Two-sided tests at 5% significance level were used in all the analyses.

## Results

### Patients

A total of 80 of 320 screened renal transplant recipients were recruited for the study between November 2008 and October 2009. Of these, 40 patients were randomly assigned to receive 7vPnC/PPV and 40 to receive PPV/PPV. After randomization, 9 patients withdrew from the study and 9 were lost to follow up, thus 62 patients patients received the second vaccination (figure S1). Demographic characteristics and other baseline characteristics of the randomized patients are shown in table 1. Vaccination was well tolerated by patients in both groups (table 2). Adverse reactions after the first immunization and after revaccination were similar in the two groups. Most reactions were local and generally mild; all were self-limited with resolution in 2 days. Three patients developed pneumonia after revaccination, but pathogen was not identified. There was no episode of allograft rejection or potential vaccine-related rejection during the 8 weeks after vaccination.

**Table 1:** Baseline characteristics of renal transplant recipients stratified for primary endpoints.

Characteristic	7vPnC/PPV (n = 33)	PPV (n = 40)	
Mean age (years ± SD)	54.05±12.04	50.47±12.80	p>0.05
Sex (M/F)	26/14	28/12	p>0.05
Time from transplant (months ± SD)	63.70±69.65	78.27±81.79	p>0.05
History of rejection (previous 6 months)	0	0	p>0.05
Serum creatinine at baseline (mean ± SD)	1.64±0.37 µmol/L	1.44±0.37 µmol/L	p>0.05
Immunosuppression at baseline:			
Corticosteroid	26 (78.8%)	28 (70%)	p>0.05
Purine synthesis inhibitor	24 (72.7%)	34 (85%)	p>0.05
Calcineurin inhibitor	28 (84.8%)	37 (92.5%)	p>0.05
Sirolimus	1 (2.5%)	2 (5%)	p>0.05
Everolimus	1 (2.5%)	1 (2.5%)	p>0.05

**Table 2:** Local and systemic reaction to vaccination.

	No (%) of patients	
Adverse event	7vPnC/PPV (n = 40)	PPV/PPV (n = 40)
local reaction	2 (5)	5 (12.5)
shivering	1 (2.5)	2 (5)
fatigue	1 (2.5)	1 (2.5)
headache	1 (2.5)	2 (5)
sweating	1 (2.5)	1 (2.5)
myalgia	2 (5)	1 (2.5)

### Immunogenicity

For the primary endpoint, serotype-specific antibody concentrations measured by ELISA were compared in the primed 7vPnC/PPV group and the PPV group. Table 3 shows the GMCs of serotype-specific IgG responses before and after a single dose of 7vPnC or PPV, and after revaccination with PPV 1 year later. Data are presented graphically in figure S2. Baseline serotype-specific IgG GMCs were high (>1.00 µg/ml) for all serotypes measured. Serotype-specific anti-pneumococcal IgG GMCs in patients after the 7vPnC vaccination were similar to those in PPV recipients. Significant increases in IgG GMCs were observed from baseline to 8 weeks following first dose for all 7vPnC serotypes in 7vPnC group and all serotypes except 6B and 19F for PPV. Decline in serotype-specific IgG GMCs was observed during the interval to revaccination, but antibody levels persisted well for the majority of serotypes in both groups. Revaccination with PPV achieved no significant increase of serotype-specific anti-pneumococcal IgG titers in the 7vPnC group when compared with the single PPV recipients (figure S3). Only modest increases were seen in IgG GMCs to serotypes 1 and 5 in the 7vPnC group following PPV. A trend for a different immune response between 7vPnC and PPV group after first vaccination was detected, but this was not significantly relevant However, for 5 serotypes (4, 6B, 14, 18C, 23F) better response was seen in the 7vPnC/PPV group than in recipients of a single dose of PPV vaccine.

**Table 3:** Geometric mean concentrations (95% CI), expressed as µg/mL, of serotype-specific anti-pneumococcal IgG serum antibody at baseline, 8 weeks, and 1 year after vaccination with 7vPnC and PPV, and 8 weeks after revaccination with PPV.

	Geometric mean titer (µg/mL)				
Serotype					
		Baseline (n = 40)	8 weeks (n = 40)	1 year (n = 62)	8 weeks after 2nd vaccination (n = 62) 7vnC/PPV (n = 33) PPV/PPV (n = 29)
4	**7vPnC/PPV**	**1.02** (0.62–1.43)	**3.61** (2.11–5.11)	**1.54** (1.06–2.03)	**3.01** (1.02–4.98)
	**PPV/PPV**	**1.21** (0.72–1.71)	**3.31** (1.98–4.65)	**2.21** (0.99–3.41)	**3.19** (1.27–5.11)
6B	**7vPnC/PPV**	**2.13** (1.54–2.74)	**8.01** (3.93–12.09)	**3.38** (2.02–4.75)	**8.11** (2.13–14.11)
	**PPV/PPV**	**3.57** (2.27–4.88)	**7.11** (4.51–9.48)	**4.62** (3.05–6.19)	**4.01** (2.98–5.72)
9V	**7vPnC/PPV**	**1.69** (1.10–2.28)	**5.38** (3.37–7.41)	**4.24** (2.76–5.73)	**4.93** (1.99–7,87)
	**PPV/PPV**	**1.94** (1.02–2.87)	**6.57** (4.33–8.82)	**5.23** (2.97–7.49)	**8.85** (4.48–13.23)
14	**7vPnC/PPV**	**8.92** (5.17–12.67)	**30.58** (18.54–42.6)	**26.05** (15.82–36.2)	**37.47** (21.35–53.56)
	**PPV/PPV**	**9.82 (**4.33–15.32)	**32.40** (21.13–43.6)	**23.01** (12.5–33.68)	**35.64** (18.25–53.02)
18C	**7vPnC/PPV**	**1.77** (1.10–2.47)	**7.08** (5.1–9.06)	**5.44** (3.53–7.35)	**5.74** (3.1–8.38)
	**PPV/PPV**	**2.43** (1.49–3.37)	**6.58** (4.3–8.56)	**4.81** (3.0–6.62)	**6.41** (3.59–9.23)
19F	**7vPnC/PPV**	**5.70** (3.55–7.84)	**9.31** (5.67–12.94)	**6.33** (3.96–8.71)	**11.83** (5.34–18.32)
	**PPV/PPV**	**5.33** (3.10–7.57)	**11.81** (7.43–16.18)	**9.05** (4.59–13.52)	**17.26** d (7.76–26.76)
23F	**7vPnC/PPV**	**3.57** (2.52–4.63)	**9.52** (6.07–12.96)	**6.72** (3.63–9.81)	**8.10** (3.59–12.61)
	**PPV/PPV**	**4.24** (2.82–5.67)	**8.77** (5.7–11.83)	**7.92** (4.3–11.54)	**12.63** (6.42–18.37)
1	**7vPnC/PPV**	**2.01** (1.48–2.54)	**2.11** (1.55–2.69)	**2.74** (1.25–4.22)	**3.41** (2.47–4.34)
	**PPV/PPV**	**2.44** (1.62–3.27)	**6.93** a (4.49–9.37)	**5.97** (3.27–8.69)	**6.62** (3.54–9.69)
5	**7vPnC/PPV**	**2.03** (1.44–2.63)	**2.18** (1.55–2.82)	**2.44** (1.37–3.52)	**2.71** (1.69–3.71)
	**PPV/PPV**	**2.08** (1.71–2.45)	**5.09** b (3.26–6.92)	**3.97** (2.1–5.84)	**3.84** (1.68–5.99)
7F	**7vPnC/PPV**	**2.95** (1.34–4.56)	**3.10** (1.46–4.71)	**2.51** (1.51–3.51)	**5.19** (2.06–8.31)
	**PPV/PPV**	**2.64** (1.65–3.63)	**7.91** c (5.61–10.22)	**5.90** (3.48–8.32)	**8.32** e (4.35–12.29)

Serotypes 1, 5, and 7F are not included in the 7vPnC vaccine.

a P<0.001 for fold increase at weeks 8 weeks, vs. 7vPNC vaccine (Mann–Whitney U Test).

b P<0.01 for fold increase at weeks 8 weeks, vs. 7vPNC vaccine (Mann–Whitney U Test).

c P<0.001 for fold increase at weeks 8 weeks, vs. 7vPNC vaccine (Mann–Whitney U Test.

d P = 0.049 for fold increase at weeks 8 weeks, vs 7vPNC vaccine (Mann–Whitney U Test).

e P = 0.004 for fold increase at weeks 8 weeks, vs 7vPNC vaccine (Mann–Whitney U Test).

We defined a vaccine response as a ≥2-fold rise in antibody concentration from baseline and an absolute post-vaccination value of at least 1 µg/ml. Comparison of vaccine responses to 7vPnC/PPV with responses to the PPV/PPV schedule 8 weeks after the second vaccination showed a significantly better response to serotypes 19F and 9F in the PPV/PPV group: 3.4 (95% CI 1.7–5.1) vs. 1.5 (95% 0.9–2.2), *P* = 0.049, and 5.6 (95% CI 3.5–7.7) vs. 3.0. (95% CI 1.5–4.4) (figure S4). A trend toward improved response in the PPV/PPV group was also seen for serotype 9V: 8.2 (95% 4.2–12.1) vs. 4.3 (95% CI 2.2–6.3), *P* = 0.056.

Figure S5 shows the number of patients with seroconversion to each serotype, defined as a minimum 2-fold titer increase and serum concentration of at least 1 µg/ml after primary vaccination and booster vaccination. After primary vaccination, the greatest number of patients showing seroconversion (≥50%) was seen for serotypes 9V, 14, 18C, 1, and 7F in the PPV group and serotype 18C in the 7vPnC group. After booster vaccination, the greatest number of patients showing seroconversion (≥50%) was for serotypes 9V, 14, and 18C in the 7vPnC/PPV group and for serotypes 9V, 18C, and 7F in the PPV/PPV group.

Eight weeks and 1 year after first vaccination, the median number of serotypes that elicited response was 3.0 (95% CI 2.3–3.7) vs. 2.0 (95% CI 1.8–3.3) in the 7vPnC group, and 5 (95% CI 3.9–6.1) vs. 3.5 (95% CI 2.7–5.0) in the PPV group. After revaccination the median number of serotypes eliciting a response was 3.5 (95% CI 2.5–4.5) in the 7vPnC/PPV group and 4.0 (95% CI 3.3–5.6) in PPV/PPV group. A response to at least 1 serotype 8 weeks after first vaccination was seen in 77.1% of patients who received 7vPnC and 93.1% of patients who received PPV (*P* = 0.046). Similar response to at least 1 serotype was seen after revaccination with PPV 1 year later: 7vPnC/PPV 87.5% vs. PPV/PPV 87.1%.

### Factors Associated with Immunogenicity

None of the candidate factors: age, time since transplant, and immunosuppression, significantly predicted response in either univariate or multivariate logistic regression modeling.

## Discussion

Current guidelines of the American Society of Transplantation state that a single dose of PPV should be given to transplant recipients, with subsequent revaccination at 3–5 years [4]. However, at present there is no evidence that PPV protects against pneumococcal infections in adults who are immunosuppressed [16].

It has been suggested by several experts that the strategy of priming with 7vPnC and boosting with PPV may be beneficial for immunocompromised patients, who may have suboptimal immunological responses to PPV alone [12], [17].

We performed a randomized, single-blind, parallel-group controlled trial of a sequential vaccination strategy involving administration of PPV after primary vaccination with either 7vPnC or PPV. Overall, both vaccination strategies were well tolerated, adverse events were generally mild, and there was no evidence of a “triggering” effect or acute rejection.

In our study, the observed immune responses to the two sequential vaccination schedules were similar. Moreover, there was no benefit in either of the sequential regimens (7vPnC/PPV, PPV/PPV) when compared with single-dose PPV vaccination as recommended by the guidelines. Significantly greater increases in antibodies to serotypes 7F and 9V were seen in the PPV/PPV group when compared with the 7vPnC/PPV group, whereas only for serotype 7F we detected a significant difference in the number of patients with seroconversion. The response to at least 1 serotype was high in both groups (7vPnC/PPV 87.5%, PPV/PPV 87.1%) and was similar to the response after primary vaccination (7vPnC 77.1%, PPV 93.1%). The previous study by Kumar et al. in liver transplant recipients showed no additional benefit in sequential pneumococcal vaccination with 7vPnC followed by PPV [9]. Further, we could not detect any benefit in an additional dose of PPV 6 weeks after 7vPnC in heart and lung transplant recipients [13].

To our knowledge there are no data on sequential vaccination in renal transplant patients. Kumar et al showed that 7vPnC is immunogenic in such patients: a response to at least 1 serotype was seen in 22 (73.3%) of 30 patients who received 7vPnC and 16 (53. 3%) of 30 patients who received PPV (*P* = 0.11) [7]. This is in contrast to our present findings (77.1% response after 7vPnC vs. 93% response after PPV; *P* = 0.046).

Several studies, however, have suggested that 7vPnC elicits a superior immune response in adults when compared with PPV [7], [18], [19]. In the majority of studies a double dose (1 mL instead of 0.5 mL pediatric dose) of the conjugated vaccine was used, thus the available data suggest that the 7vPnC vaccine, particularly when administered at twice the usual pediatric dose, elicits a more robust functional immune response in adults [16].

With regard to vaccine response to at least 1 serotype, our finding of reduced response after a single dose of 7vPnC vaccine, compared with the PPV group, may be due to the low dose (0.5 mL) of 7vPnC used in the present study. The dose of serotype-specific antigen in PPV is 6–10 times greater than that contained in 7vPnC (25 µg vs. 2 µg or 4 µg) [20], and the greater antigen dose may contribute to the higher antibody concentrations observed. Nevertheless, the quality of the antibodies, such as avidity or opsonic functional capacity, could not be determined by ELISA and thus we do not know whether patients with increased antibody concentrations show greater clinical protection from invasive pneumococcal disease.

Some controlled studies in the non-transplantation context have shown the potential benefit of a combined vaccination schedule, mainly in children. In adults, however, the results are more conflicting.

In agreement with our results, other recent studies using a second dose of vaccine in adults have failed to show that the initial dose of 7vPnC induces a boostable or memory response. It is unclear why we are unable to demonstrate boostability after a dose of 7vPnC in adults as we do in infants [21]–[24]. Previously, Baxendale et al [25] showed that on day 7 after immunization of healthy young adults with 7vPnC or PPV, pneumococcal-specific B cells with all the characteristics of memory B cells could be isolated, suggesting that memory had been established before vaccination [26]. It is possible that pneumococcal antigen-experienced adults who have encountered *S. pneumoniae* in the nasopharynx develop immunologic memory and, when they are immunized with a first dose of 7vPnC or PPV, existing memory cells are stimulated.

The time period between the prime and the boost vaccine may be important. In a study by de Roux et al in elderly patients ≥70 years of age, a combined vaccination schedule of 7vPnC followed 1 year later by PPV induced a significant increase in immune response after the revaccination with PPV [7]. Similarly, in a study by Chan et al, 39 patients with Hodgkin lymphoma, who were first immunized with 7vPnC and revaccinated with PPV 1 year later, were able to mount significantly higher serotype-specific antibody concentrations than patients who received PPV alone [27]. In contrast, in the study by Goldblatt et al no benefit could be observed after a second vaccination with either PPV or 7vPnC in older patients receiving a 7vPnC 6 months previously. Further, Miernyk et al found no improved immune response in native Alaskan adults given 7vPnC 2 or 6 months before PPV [28], and neither Kumar et al nor Gattringer et al could demonstrate any benefit of a booster vaccine 6–8 weeks after the prime vaccine in liver, heart or lung transplant recipients [13], [29]. Moreover, Musher et al recently reported that subjects who received PPV within a year of prior vaccination had almost no response to the revaccination, although IgG levels increased in proportion to the time elapsed after the first vaccination [30]. On the basis of those results we used a time period of 1 year between the first and second vaccinations in the present study and did not observe any benefit of a second vaccination with PPV 1 year after priming with 7vPnC.

We compared two sequential vaccine regimes, 7vPnC/PPV and PPV/PPV, with single PPV vaccination. The immune response to capsular polysaccharides is T cell independent and should have a limited duration. If waning antibody levels are associated with decreased clinical protection, then it is reasonable to conclude that revaccination may be appropriate.

The American Advisory Committee on Immunization Practices (ACIP) of the Centers for Disease Control and Prevention recommends that all persons ≥65 years old receive PPV and that a single revaccination be administered if ≥5 years have elapsed since the initial dose and if they were <65 years old at time of primary vaccination. Repeated vaccination has been shown to be relatively free of adverse events [31].

Elderly persons who have been revaccinated with PPV show significant antibody responses to most serotypes studied [32]. However, the magnitude of the antibody response in some studies has been lower than after initial vaccination, raising questions about whether these adults may experience hyporesponsiveness or immune tolerance to repeated doses of these polysaccharide antigens [20].

In recent studies by Musher et al and Manoff et al, primary vaccination and revaccination within 3–5 years with PPV induced antibody response in middle-aged and older adults [30], [33] without evidence of significant hyporesponsiveness with revaccination. Nonetheless, as mentioned above, Musher et al reported that subjects who had received PPV within a year of prior vaccination had almost no response to revaccination with PPV, indicating that this time interval may be too short and may induce hyporesponsiveness. In contrast, Landgren et al reported that splenectomized individuals with Hodgkin lymphoma showed improved immune responses after repeated PPV vaccination beginning 1 year after the initial PPV dose [34]. In our study lower response after revaccination for serotypes 4 and 6B in PPV/PPV group and for serotypes 4, 9V, 18C and 23F in 7vPnC/PPV group was detected. It is unclear why hyporesponse to second vaccination occurs for these serotypes.

There are limited data on longitudinal follow-up of pneumococcal antibody levels in organ transplant recipients. McCashland et al vaccinated patients with chronic liver disease who went on to receive a liver transplant. Pneumococcal antibody levels declined to baseline levels rapidly in the first few months post-transplant [35]. In a study by Blumberg et al [5], 7 heart transplant recipients showed a decline in antibody titer to 50–80% over 2 years. In renal transplant recipients, Kumar et al found a significant decline of vaccine response 3 years after vaccination and use of conjugate vaccine did not improve the durability of response [36]. However, it was not shown how quickly the antibodies decline in such high risk patients. In an earlier study in 33 renal transplant recipients, patients were found to have significantly waning antibody levels 2 years post-polysaccharide vaccine [37], [38].

In the present study we measured antibody concentrations 1 year after vaccination with 7vPnC or PPV and found that serotype-specific IgG titers decreased after both vaccine regimes. However, in comparison with baseline, no significant difference was detected in antibody response 8 weeks and 1 year after vaccination with single dose of 7vPnC or PPV.

On the basis of the above data, in the present study we also revaccinated the PPV group 1 year after the initial PPV dose to evaluate whether this approach might show a benefit in immune response. Our results showed that the PPV/PPV regimen leads to no significant improvement of the immune response when compared with single PPV vaccination.

Although many of the diseases that routine immunization prevents are rare in recipients of solid organ transplants, it is important that these patients remain up to date with their immunizations. As result of immunosuppression, antibody titers achievable in transplant candidates are often suboptimal and, associated with the underlying liver or renal failure, antibody responses are usually even worse when vaccines are given after transplantation. However, immunosuppressive therapy is continually being improved and in most cases little is known about the impact of the more recently adopted regimes on durability of antibody response. We found that use of corticosteroid or use of triple therapy comprising glucocorticoids (prednislone), calcineurin inhibitors (cyclosporine or tacrolimus), and purine synthesis inhibitors (mycophenolate mofetil, azathioprine) did not affect the immune response. The 7vPnC vaccine is T cell dependent and stimulates production of memory B cells. In the present study, although we hypothesized that memory B cells may be further stimulated by a polysaccharide booster to produce higher antibody titers than observed with administration of the standard single-dose PPV, no benefit of revaccination could be detected.

A limitation of our study is that we used anti-pneumococcal IgG antibodies measured by ELISA as a surrogate marker for vaccine efficacy. However, there is no consensus regarding the antibody level that provides protection against infection in adults, nor what defines an appropriate vaccine response [16]. Although older adults develop antibody titers that are similar to those in their younger counterparts, these antibodies have reduced function [39] and ELISA cannot distinguish between functional and nonfunctional antibodies [40]. A further limitation of our study is that we did not measure in vitro opsonophagocytic killing (OPK) activity of serum antibodies with a phagocytic cell line. OPK has been shown to correlate with immune protection in animal studies [41] but there have been no studies correlating OPK assay results with protection in adults. Furthermore, unlike ELISA, the available 7-serotype opsonophagocytic assay is not standardized or validated.

We were also limited by the relatively small sample size of patients and by 18 of the 80 (22.5%) patients dropping out of the study, a higher drop-out rate than expected. The logistic regression analysis should therefore be regarded as exploratory, since the power to detect variables of significance is limited.

In conclusion, although the strategy of revaccination 1 year later after primary vaccination did not cause any significant adverse events, such as rejection, our results suggest no additional benefit of sequential pneumococcal vaccination with 7vPnC followed by PPV in adult recipients of renal transplants. Although a stronger immune response was seen in the PPV/PPV group than in the 7vPnC/PPV group, significant increase in antibody response was seen for two serotypes only.

## Supporting Information

Figure S1 **Flow chart.**
(DOCX)

Figure S2 **Geometric mean concentration (GMC) of 10 serotype-specific antipneumococcal IgG serum antibodies at baseline (A), 8 weeks (B) and 1 year (C) after vaccination with 7vPnC and PPV, and 8 weeks after revaccination with PPV (D).** Note that the Y-axis scale for serotype 14 differs from the other serotypes (with 95% confidence interval). Serotypes 1, 5, and 7F are not included in the 7vPnC vaccine.
(TIF)

Figure S3 **Fold increase of antibody response in the 7vPnC/PPV group 8 weeks after revaccination compared with the immune response of the PPV group 8 weeks after vaccination.** Fold increase in ELISA concentrations were determined by dividing the post-vaccination concentrations by the pre-vaccination concentrations in each group.
(TIF)

Figure S4 **Fold increase of antibody response compared with baseline 8 weeks after revaccination with PPV.** Fold increase in ELISA concentrations were determined by dividing the final post-vaccination concentrations by the pre-vaccination concentrations in each group.
(TIF)

Figure S5 **Percentage of subjects responding to each serotype 8 weeks after vaccination on the basis of serotype-specific antibody response.** Serotypes 1, 5, and 7F are not included in the 7vPnC vaccine. a) after first vaccination; b) after second vaccination
(TIF)

Checklist S1 **CONSORT 2010 checklist of information to include when reporting a randomised trial.**
(DOC)

Protocol S1 **Trial Protocol.**
(DOC)
