# Supplementary material for: Randomized, Single Blind, Controlled Trial to Evaluate the Prime-Boost Strategy for Pneumococcal Vaccination in Renal Transplant Recipients
Source: PLoS One. 2012 Sep 28;7(9):e46133. doi: 10.1371/journal.pone.0046133 (PMC3460962; doi:10.1371/journal.pone.0046133)
Supplement: Figure S1 — Flow chart. (DOCX) [file pone.0046133.s001.docx]

Lost to follow up (n=4)

Withdrew from study (n=3)

Lost to follow up (n= 5)

Withdrew from study (n=6)

Allocated to PPV/PPV (n=40)

Received allocated intervention with PPV (n=40)

Allocated to 7vPnC/PPV (n=40)

Received allocated intervention with 7VPn (n=40)

## Follow-Up

Primary vaccination (n=40)

Secondary vaccination (n=33)

## Analysis

Primary vaccination (n=40)

Secondary vaccination (n=29)

## Enrollment

## Allocation

Randomized (n=80)

Excluded (n=220)

♦  Not meeting inclusion criteria (n=100)

♦  Declined to participate (n=110)

♦

Assessed for eligibility (n= 300)
