# Supplementary material for: Randomized, Single Blind, Controlled Trial to Evaluate the Prime-Boost Strategy for Pneumococcal Vaccination in Renal Transplant Recipients
Source: PLoS One. 2012 Sep 28;7(9):e46133. doi: 10.1371/journal.pone.0046133 (PMC3460962; doi:10.1371/journal.pone.0046133)
Supplement: Protocol S1 — Trial Protocol. (DOC) [file pone.0046133.s007.doc]

Allgemeines Krankenhaus

Department of

Internal Medicine I

Division of Infectious Diseases and Tropical Medicine

Medical University Vienna

_______________________________________________________________________________________________________________

Währinger-Gürtel 18-20

A-1090 Vienna

Austria

Tel.: + 43 1 40400 4440

Fax.: + 43 1 40400 4418

email:rainer.gattringer@meduniwien.ac.at

|  |  |
| --- | --- |

Study title:

**Determination of immune response after sequential pneumococcal vaccination using the 7-valent conjugate pneumococcal- and the 23-valent pneumococcal polysaccharide vaccine in renal transplant recipients**

**Date: August 1^st^ , 2007**

CLINICAL STUDY PROTOCOL

Protocol Authors: Heinz Burgmann, MD

Rainer Gattringer, MD

Co-Investigators: Stefan Winkler, MD

Heidi Winkler, MT

Gere Sunder Plassmann, MD

Principal Investigator according

AMG (AMG §§ 2a, 35, 36): Heinz Burgmann, MD

Department of Internal Medicine I

Division of Infectious Diseases and Tropical Medicine

Sponsor (AMG §§ 2a, 31, 32): Department of Internal Medicine I

Division of Infectious Diseases and Tropical Medicine

Monitor (AMG §§ 2a, 33, 34): Heimo Lagler, MD

Department of Internal Medicine I

Division of Infectious Diseases and Tropical Medicine

Univ. Prof Dr. Gere Sunder-Plassmann is working at Department of Internal Medicine III, Division of Nephrologie, all other investigators are working at the Department of Internal Medicine I, Division of Infectious Diseases and Tropical Medicine, Medical University Vienna.

## Study synopsis

##

**Title:** Determination of immune response after sequential pneumococcal vaccination using the 7-valent conjugate pneumococcal- and the 23-valent pneumococcal polysaccharide vaccine in renal transplant recipients.

**Background:** Although many of the diseases that routine immunization prevents are rare in solid organ transplant recipients, there are several important reasons to ensure that these patients remain up to date on their immunizations. First, while antibody titers achievable in transplant candidates are often suboptimal as a result of immunosuppression. Associated with the underlying liver or renal failure, antibody responses are usually even worse when vaccines are administered after transplantation. Second, the safety of immunization with living virus vaccine such as MMR and varicella is not established, so that these vaccines are contraindicated in transplant recipients receiving exogenous immunosuppression. Third, many transplant recipients are not immunized after transplantation because of concerns about allograft rejection triggered by immunization.

The challenges to immunize solid organ transplant candidates include unpredictable waiting times, requiring accelerated schedules of vaccine administration in some patients while others wait for prolonged periods and require regular re-evaluation of their immunization status.

In addition, immunosuppressive therapy is being improved continually and in most cases little is known about the impact of the more recently adopted regimens on durability of antibody response. Thus studies on immune-response after vaccination in patients with different immunosuppressive regimes are still missing.

*Streptococcus pneumoniae*, a common pathogen colonized in the upper respiratory tract, is a cause of substantial morbidity and mortality by causing pneumonia, otitis media, sinusitis and invasive infections, such as bacteremia and meningitis. The risk of invasive pneumococcal disease among renal transplant recipients is about 1%/year or 28 infections/1000 patient years – this is > 60 times higher than the rate in the general population.

Pneumococcal polysaccharide vaccine (PPV23), a 23-valent vaccine derived from capsular polysaccharides of *S. pneumoniae*, is widely used and is recommended by WHO for all individuals exceeding the 60^th^ and the 65^th^ year of life, respectively, and for those with underlying chronic illness or pre- and post-splenectomy. While the efficacy of the vaccine has been discussed controversial several controlled studies showed a reduction of community acquired pneumonia and a reduction of mortality from pneumonia after vaccination. On the other hand Jackson et al found that while bacteremia can be clearly reduced in patients having received the vaccine, there was no overall reduction of pneumonia of any cause. Taken together the PPV23 vaccine appears to provide benefits but also has limitations.

There is little information on the safety and efficacy of the polysaccharide pneumococcal vaccine in transplant recipients. Antibody concentrations decline substantially 2 years after immunization, but revaccination leads to a modest increase in antibody levels.

However, the use of immunsuppressive medications such as cyclosporine, prednisolon, tacrolimus and mycophenolate mofetil may impair these patients response to vaccination. Studies that assessed the immunogenicity of polysaccharide vaccine in renal transplantation have demonstrated variable protective efficacy but, collectively, have produced suboptimal responses when functional antibody responses have been assessed. One way to overcome this problem is the development of more effective vaccines.

Recently, conjugate pneumococcal vaccines have been developed, whereby the immunogenicity of capsular polysaccharides is enhanced by covalent conjugation with carrier proteins. The currently available conjugate pneumococcal vaccine (PCV7) contains 7 capsular polysaccharides (serotype 4, 6B, 9V, 14, 18C, 19F and 23F).

The efficacy of the conjugate vaccine is due to several mechanisms: increased amounts of circulating antibodies, higher avidity, and an induction of immunological memory.

In a randomized trial comparing PPV23 to PCV7 in adult kidney transplant recipients, it could be demonstrated that the conjugate vaccine is immunogenic in this population. However, there was no significant difference in immunogenicity demonstrated between the two vaccines at 8 weeks postvaccination.

It could also be shown that the conjugate vaccine does not lead to a more durable antibody response for any of the 7-serotypes covered in this vaccine when compared to polysaccharide vaccine. Overall response rates were generally suboptimal for both groups, but this could potentially be overcome with repeat dosing schedules or higher doses of vaccine.

Booster vaccinations may be performed by using the conjugated vaccine several times or – as described in the literature by using the conjugate vaccine to prime the immune system and then give a booster dose of the polysaccharide vaccine. This may result in an even greater response than a single vaccine dose and has shown promise in some groups of immunocompromised patients.

Overall the development of a polysaccharide-specific memory seems to be influenced by the age at priming with the glycoconjugate, the route of immunization, the time and features of the booster, and other factors.

However, the combination of an initial series of conjugate vaccine followed by administration of the 23-valent polysaccharide vaccine is recently recommended by American Academy of Pediatric Committee on Infectious Diseases and the Robert Koch Institute for patients with solid organ transplantation.

For pediatrics Ling Li et al tested the pneumococcal sequential vaccination schedule in heart transplant recipients recommended by the American Academy of Pediatrics. It consists of 2 doses of PCV7 and a single dose of PPV23. Each vaccine dose was given 2 month apart. Controls received a single dose of PCV7 followed 2 month later by a single dose of PPV23. Significant rises in serotype-specific pneumococcal antibody geometric mean concentrations from prevaccination levels were observed in both groups. Antibody concentrations did not increase significantly among solid organ transplant patients after the second dose of PCV7. No additional increase in PCV7 associated serotype-specific antibody levels was observed after PPV23 dose in both groups. The authors conclude, that solid organ transplant recipients do not benefit from the second dose of PCV7 or from the PPV 23 dose given 2 months later.

As already stated the time interval between PCV7 and PPV23 should be > 2 months.

Patients with SOT may benefit from a single dose of PCV7 followed by PPV23 12 months later. Children who have received PCV7 previously as part of the childhood immunization program might respond better if the PPV23 is given at least 12 month after the last dose of PCV7.

**Aims of the study:** The aim of this study is to compare the quantitative antibody responses of a sequential vaccination schedule of PPV23 with those of a sequential schedule of PCV7 followed by PPV23 12 months later in adult renal transplant recipients by measuring the concentrations of serotype-specific IgG in sera using an isotype-specific ELISA that has recently been recommended by the World Health Organization.

For a subset of subjects the T-cell mediated immune response will be evaluated in both groups.

**Study design:** randomized, double blind, controlled trial

**Methods:** Taking blood to quantify specific immunoglobulins und cellular immunity.

**Study drugs** *1. PPV23* (Pneumo 23®)

1. *PCV23* (Prevenar ®)

**Subjects:** A total of 78 renal transplant patients will

be enrolled into the study.

**Main outcome variable:** 1. Antibody concentrations after vaccination

- Fold increase of antibody response as compared to baseline 8 weeks after the vaccination

- Number of subjects with seroconversion defined as minimum 2-fold titer increase and a serum concentration of at least 1 µg/ml

- Number of subjects with at least > 2-fold increase for 2 of the 7 measured serotypes

2. For the subset of subjects included in the evaluation of cellular immunity

- T-cell response after each vaccination as determined by the frequency of cytokine producing T-cells induced by conjugated pneumococcal Ag

- Increase in frequency of cytokine producing T-cells induced by conjugated pneumococcal Ag after each vaccination as compared to baseline

.

**Statistics:** All patients will be included in the analysis as randomised.

Although the primary endpoint is the difference in antibody concentration as continuous variable between study groups we base our sample size calculation on the more conservative but clinically more useful binary variable vaccine response. A vaccine response by ELISA will be defined as a > 2 fold increase in prevaccination titer and an absolute postvaccination value of at least 1 µg/ml.

The study is powered to detect an improvement in vaccine response with the conjugate vaccine in any of the 7 serotypes. The study is set to yield a power of 80% at a significance level of 5% to detect a 35% improvement in the response rate for a given serotype. The response rate in the control group is expected at about 45%. Thus the calculated sample size will be 35 in each group. We added 10% to adjust for potential loss-to-follow-up which results in 39 patients in each group.

We expect antibodies being log-normally distributed. Geometric mean antibody concentrations (GMCs) for each pneumococcal serotype will be compared between the two groups by using the 2-tailed, unpaired Students’s t-test after log transformation of the raw data, given our distributional assumptions hold.

For binary variables we will calculate risk ratios and exact 95% confidence intervals. Vaccination efficacy (E_v_) will be calculated as: E_v_ = (1-RR) x 100. For hypothesis testing we will use the Fisher’s exact test.

All analysis will be conducted by using a 2-sided p value with a type I error rate of < 0.05.

**Safety precautions:** Patients will be instructed to record local reactions /e.g. redness, swelling, and tenderness) and systemic reactions (e.g. fever, nausea, and vomiting).

At the time of vaccination, medical providers will be asked to record appearance of local reactions occurring within 30 minutes of injection. At the time of each vaccination visit patients will be given a diary card, thermometer and a measuring tape. On postvaccination days 1 through 3 patients will be asked to record presence or absence of redness and swelling (in millimetres), pain/tenderness (graded none, mild, moderate or severe), loss of appetite, vomiting, headache, fussiness, increased sleep or other potential adverse reactions. Data from the diary will be recorded as well as changes in medications or medical care.

# **Scientific/Medical section**

**Introduction:**

Although many of the diseases that routine immunization prevents are rare in solid organ transplant recipients, there are several important reasons to ensure that these patients remain up to date on their immunizations. First, while antibody titers achievable in transplant candidates are often suboptimal as a result of immunosuppression associated with the underlying liver or renal failure, antibody responses are usually even worse when vaccines are administered after transplantation. Second, the safety of immunization with living virus vaccine such as MMR and varicella is not established, so that these vaccines are contraindicated in transplant recipients receiving exogenous immunosuppression. Third, many transplant recipients are not immunized after transplantation because of concerns about allograft rejection triggered by immunization.

The challenges to immunize solid organ transplant candidates include unpredictable waiting times, requiring accelerated schedules of vaccine administration in some patients while others wait for prolonged periods and require regular re-evaluation of their immunization status.

For tetanus and diphtheria we evaluated the immune status in patients with chronic heart failure and after heart transplantation. As seen in the figure the immunisation rate for diphtheria (% of patients with serum concentrations above the so called protective value) is poor and revaccination should be performed in both groups of patients. Tetanus vaccine is a vaccination which is used frequently in Austria, therefore adequate antibody titers are even found in such “risk patients”.

Solid organ transplant recipients usually receive lifelong immunosuppression. No formal recommendations have been developed about when to resume immunizations but many transplant centers withhold immunizations until the patients is taking baseline immunosuppression. In some centers, all immunizations are withheld during the first transplant year. In the present study we will start with immunization at least 6 months after transplantation.

In addition, immunosuppressive therapy is being improved continually and in most cases little is known about the impact of the more recently adopted regimens on durability of antibody response. Thus studies on immune-response after vaccination in patients with different immunosuppressive regimes are still missing.

*Streptococcus pneumoniae*, a common pathogen colonized in the upper respiratory tract, is a cause of substantial morbidity and mortality by causing pneumonia, otitis media, sinusitis and invasive infections, such as bacteremia and meningitis. The risk of invasive pneumococcal disease among renal transplant recipients is about 1%/year or 28 infections/1000 patient years – this is > 60 times higher than the rate in the general population. (Fedson, Linnemann CC).

Pneumococcal polysaccharide vaccine (PPV23), a 23-valent vaccine derived from capsular polysaccharides of *S. pneumoniae*, is widely used and is recommended by WHO for all individuals exceeding the 60^th^ and the 65^th^ year of life, respectively, and for those with underlying chronic illness or pre- and post-splenectomy. While the efficacy of the vaccine has been controversial (Butler et al, Ortqvist et al.) several controlled studies showed a reduction of community acquired pneumonia any cause and a reduction of mortality from pneumonia after vaccination (Gaillat et al, Nichol et al.). On the other hand Jackson et al. found that while bacteremia can be clearly reduced in patients having received the vaccine, there was no overall reduction of pneumonia of any cause. Taken together the PPV23 vaccine appears to provide benefits but also has limitations.

In a previous study we could demonstrate that patients with splenectomy and haematological malignancies responded poorly to the PPV23. (Eigenberger et al.)

Figure:

Log-ratio of antibody concentrations before and after vaccination with PPV23 and Haemophilus type b PRP vaccine in splenectomised patients with haematological malignancies or trauma *P < 0.001


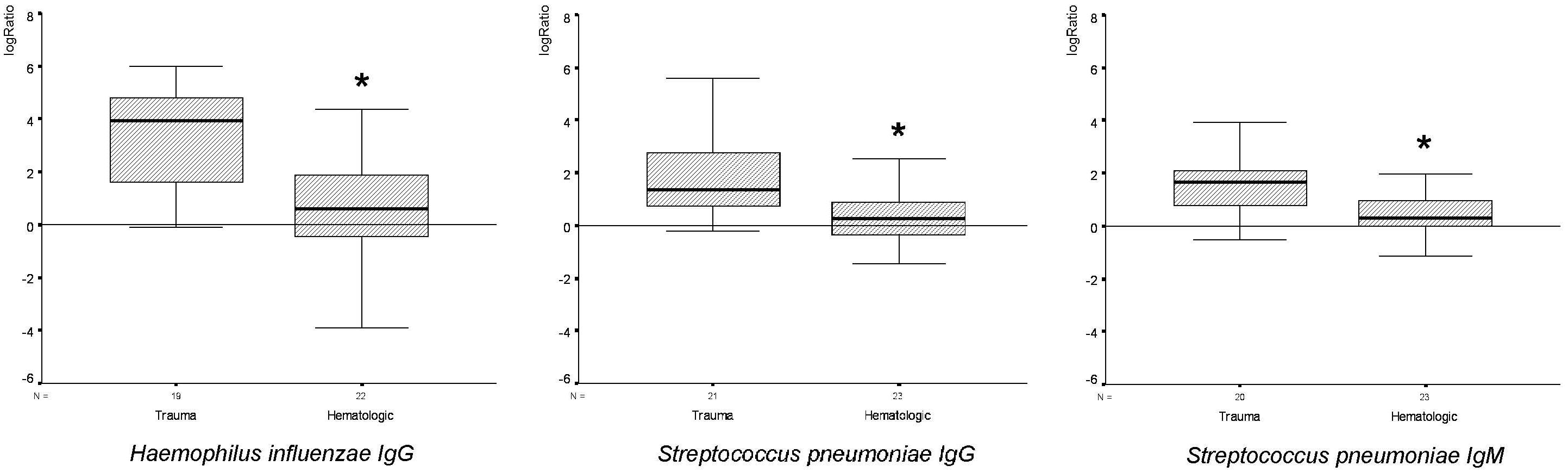


We concluded, that antibody-measurements after vaccination are mandatory in high risk patients.

There is little information on the safety and efficacy of the polysaccharide pneumococcal vaccine in transplant recipients. Antibody concentrations decline substantially 2 years after immunization, but revaccination leads to a modest increase in antibody levels.

However, the use of immunsuppressive medications such as cyclosporine, prednisone, tacrolimus and mycophenolate mofetil may impair these patients response to vaccination. Studies that assessed the immunogenicity of polysaccharide vaccine in renal transplantation have demonstrated variable protective efficacy but, collectively, have produced suboptimal responses when functional antibody responses have been assessed. One way to overcome this problem is the development of more effective vaccines.

Recently, conjugate pneumococcal vaccines have been developed, whereby the immunogenicity of capsular polysaccharides is enhanced by covalent conjugation with carrier proteins. The currently available conjugate pneumococcal vaccine (PCV7) contains 7 capsular polysaccharides (serotype 4, 6B, 9V, 14, 18C, 19F and 23F).

The efficacy of the conjugate vaccine is due to several mechanisms: increased amounts of circulating antibodies, higher avidity, and an induction of immunological memory. (Ahmed, Eskola-Antilla, Jodar, Chan,)

PCV7 elicits a T-cell dependent response, which, in turn, induces the T helper cell to stimulate polysaccharide-specific B cells to mature into either antibody-producing plasma cells or memory cells (Eskola J, Stein). The creation of this immunologic memory may be crucial in immunocompromised organ transplant recipients, in whom immunity to pneumococcus wanes quickly after polysaccharide vaccination. (Linnemann, First).

In a randomized trial comparing PPV23 to PCV7 in adult kidney transplant recipients, it could be demonstrated that the conjugate vaccine is immunogenic in this population. However, there was no significant difference in immunogenicity demonstrated between the two vaccines at 8 weeks postvaccination.

It could also be shown that the conjugate vaccine does not lead to a more durable antibody response for any of the 7-serotypes covered in this vaccine when compared to polysaccharide vaccine. Overall response rates were generally suboptimal for both groups, but this could potentially be overcome with repeat dosing schedules or higher doses of vaccine.

Additionally we could demonstrate in asplenic patients with haematological malignancies that an adequate immunogenic response, defined in our study by a 2-fold increase or more of antibodies against each single serotypes included in the pneumococcal conjugate vaccine, cannot be expected after single-shot vaccination. (Gattringer et al. submitted) Thus, as mentioned above, it is suggested that booster vaccinations may be necessary.

Table. Percentage (number) of patients with an antibody response *lower* than two-fold to single serotypes. (HM haematological malignancy, T trauma, L lymphoma)

| **Serotypes**  **Patients**  **(n = number**  **of patients**) | **4** | **6B** | **9V** | **14** | **18C** | **19F** | **23F** |
| --- | --- | --- | --- | --- | --- | --- | --- |
| **HM – group**  **(n = 24)** | 58.3%  (n = 14) | 70.8%  (n = 17) | 45.8%  (n = 11) | 58.3%  (n = 14) | 41.7%  (n = 10) | 75%  (n = 18) | 50%  (n = 12) |
| **T – group**  **(n = 28)** | 39.3%  (n = 11) | 53.6%  (n = 15) | 42.9%  (n = 12) | 60.7%  (n = 17) | 57.1%  (n = 16) | 67.9%  (n = 19) | 42.9%  (n = 12) |
| **LY – patients**  **(n = 18)** | 61.1%  (n = 11) | 72.2%  (n =13) | 44.4%  (n = 8) | 55.6%  (n = 10) | 38.9%  (n = 7) | 72.2%  (n = 13) | 50%  (n = 9) |
| **Vaccinated before splenectomy**  **(n = 18)** | 16.6%  (n = 3) | 33.3%  (n = 6) | 33.3%  (n = 6) | 38.9%  (n = 7) | 38.9%  (n = 7) | 44.4%  (n = 8) | 33.3%  (n = 6) |
| **Vaccinated after splenectomy**  **(n = 34)** | 64.7%  (n = 22) | 76.5%  (n = 26) | 50%  (n = 17) | 64.7%  (n = 22) | 55.9%  (n = 19) | 85.3%  (n = 29) | 52.9%  (n = 18) |
| **Age > 60 years**  **(n = 21)** | 61.9%  (n = 13) | 66.6%  (n = 14) | 47.6%  (n = 10) | 61.9%  (n = 13) | 52.3%  (n = 11) | 71.4%  (n = 15) | 61.9%  (n = 13) |
| **Age < 60 years**  **(n = 31)** | 38.7%  (n = 12) | 58.1%  (n = 18) | 41.9%  (n = 13) | 58.1%  (n = 18) | 48.3%  (n = 15) | 71%  (n = 22) | 35.5%  (n = 11) |
| **HM-group**  **Vaccinated before splenectomy**  **(n = 7)** | 28.6%  (n = 2) | 57.1%  (n = 4) | 42.9%  (n = 3) | 57.1%  (n = 4) | 42.9%  (n = 3) | 57.1%  (n = 4) | 57.1%  (n = 4) |
| **HM-group**  **Vaccinated after splenectomy**  **(n = 17)** | 70.6%  (n = 12) | 76.5%  (n = 13) | 47.1%  (n = 8) | 58.8%  (n = 10) | 41.2%  (n = 7) | 82.4%  (n = 14) | 47.1%  (n = 8) |
| **T – group**  **Vaccinated before splenectomy**  **(n = 11)** | 9.1%  (n = 1) | 18.2%  (n = 2) | 27.3%  (n = 3) | 36.4%  (n = 4 ) | 45.5%  (n = 5) | 45.5%  (n = 5) | 18.2%  (n = 2) |
| **T – group**  **Vaccinated after splenectomy**  **(n = 17)** | 58.8%  (n = 10) | 76.5%  (n = 13) | 52.9%  (n = 9) | 70.6%  (n = 12) | 70.6%  (n = 12) | 82.4%  (n = 14) | 58.8%  (n = 10) |

Booster vaccinations may be performed by using the conjugated vaccine several times or – as described in the literature by using the conjugate vaccine to prime the immune system and then give a booster dose of the polysaccharide vaccine. This may result in an even greater response than a single vaccine dose and has shown promise in some groups of immunocompromised patients (Vernacchio L, Chan CY, Feikin DR). However, in adults it is not known how many booster vaccinations with the PCV7 are necessary before using the PPV23 vaccine. As shown in a previous publication by Rose et al. PCV7 repeated priming has the potential to support a booster response, improving immunogenicity and efficacy.

It could be shown in adult patients with specific antibody deficiency (SPAD) which are unable to respond to PPV23 that vaccination with the conjugated vaccine can enforce a response (Shrimpton et al). However, only repeated immunization with the PCV7 leads to an adequate response, whereas a single immunization with the conjugated Hemophilus influenzae type b vaccine gives a robust response. It is a present unclear why the Hib conjugate vaccine induces a brisk response after a single injection, while the PCV7 requires repeated immunization.

In a publication by Vernacchio et al (2000), 23 children and young adults with sickle cell disease receive either a single dose of a PPV23 or 2 doses of PCV7 8 weeks apart followed 8 weeks later by 1 dose of PPV23. IgG geometric mean pneumococcal antibody concentrations measured by ELISA were higher in the PCV7-PCV7-PPV23 group, however no significance was reached. Using the flow cytometric opsonophagocytic assay titers were higher in the PCV7-PCV7-PPV23 group reaching significance for 6B, 14, 18C, 19F and 23F.

In a publication by Stoehr et al 39 splenectomised patients with hereditary sperocytosis received one dose of PCV7 and 2 month later one dose of PPV23. Eight weeks after PCV7 all subjects showed significant increase of their serum GMCs for all PCV-7 contained serotypes. Four weeks after PPV23 specific pneumococcal antibodies for serotypes 5 and 7 also increased significantly from initial values- for other serotypes no further significant increase was observed. The authors conclude, that the time between PCV7 and PPV23 seems to have an impact on the antibody response and should be at least 12 months.

In a publication by Rose et al. children (2-18 years) previously non-responders to PPV23 received two doses of PCV7 in a 4-6 week interval and a booster dose of PPV23 1 year after the first PCV7 dose (group A). The control group received one dose of PPV23 and another dose of PPV23 1 year later without previous PCV priming (Group B). After PCV children showed significantly higher antibody concentrations for all PCV7 containing serotypes already 7 days after booster vaccination. Additionally of group A 64-100% had antibody concentrations of > 1µg/ml on day 28 after the booster versus 25 to 94% of group B.

Two other studies in healthy senior subjects, however, could not find a benefit from a PPV23 booster 1 (Shelly, Jacoby) or 6 months (Powers) after PCV7 immunization. The subsequent administration provided no additional antibody response. Similarly, a small study of adults receiving a second dose of a similar pneumococcal conjugate vaccine did not demonstrate a marked improvement in antibody titers (Shelly et al).

Overall the development of a polysaccharide-specific memory seems to be influenced by the age at priming with the glycoconjugate, the route of immunization, the time and features of the booster, and other factors.

However, the combination of an initial series of conjugate vaccine followed by administration of the 23-valent polysaccharide vaccine is recently recommended by American Academy of Pediatric Committee on Infectious Diseases and the Robert Koch Institute for patients with solid organ transplantation. (Epidemiologisches Bulletin).

For pediatrics Ling Li et al tested the pneumococcal sequential vaccination schedule in heart transplant recipients recommended by the American Academy of Pediatrics. It consists of 2 doses of PCV7 and a single dose of PPV23. Each vaccine dose was given 2 month apart. Controls received a single dose of PCV7 followed 2 month later by a single dose of PPV23. Significant rises in serotype-specific pneumococcal antibody geometric mean concentrations from prevaccination levels were observed in both groups. Antibody concentrations did not increase significantly among solid organ transplant patients after the second dose of PCV7. No additional increase in PCV7 associated serotype-specific antibody levels was observed after PPV23 dose in both groups. The authors conclude, that solid organ transplant recipients do not benefit from the second dose of PCV7 or from the PPV 23 dose given 2 months later.

As already stated the time interval between PCV7 and PPV23 should be > 2 months.

Patients with SOT may benefit from a single dose of PCV7 followed by PPV23 12 months later. Children who have received PCV7 previously as part of the childhood immunization program might respond better if the PPV23 is given at least 12 month after the last dose of PCV7.

**Immune response**

Current screening methods for evaluating vaccines rely heavily on serologic responses. It could be shown by McElhaney et al. that in adults aged 60 years and older pre- and postvaccination antibody titers after influenza vaccination did not distinguish between subjects who would subsequently develop influenza illness and those who would not (non-LDI-subjects). In contrast, PBMC restimulated ex vivo with live influenza virus preperations showed statistically significant differences between LDI and non-LDI subjects. The mean INFgamma:IL10 ratio in influenza stimulated PBMC was 10-fold lower in LDI vs. non-LDI subjects. The lower ratio in LDI subjects reflects a shift toward a TH2 response and is a potential marker of continued risk of influenzas despite vaccination.

Cellular immunity has rarely been tested in context with infectious agents and vaccination studies. Recent assays for measuring human cellular immunity in clinical trials, eg. INFgammaELIspot assay, and intracellular cytokine measurement by flow cytometry have significantly increased the feasibility to study T-cell responses in clinical trials. (Lindemann, Knutson, Avetisyan)

Kamboj et al. used ELISPOT analysis to measure memory T cell cytokine production after vaccination of adults with conjugated Hib-CRM197 Hemophilus influenzae vaccination. Hib-CRM197 induced a substantial increase in both Th1 (IL2 and IFN-gamma) and Th2 (IL5 and IL10) secreting T cells.

In another publication Kamboj et al showed that immunization with the PCV7 induces a mixed Th1/Th2 CD4+ T cell cytokine response. Immunization with the PCV7 yielded an increase in PBMCs that secreted IL2, IL4, IL5, IL6, IL10 and INF-gamma within 7 days after immunization. The cytokines produced were a direct result of recall responses by carrier portein-specific T cells after reexposure to carrier protein because only CRM197 not polysaccharide or control protein induced T-cell response. In contrast, PBMCs from control subjects immunized with PPV23 produced only small quantities of IL6. This T cell memory may enhance protection induced by vaccines.

Although the reasons for improved effect of conjugate vaccine are not entirely understood, the T lymphocytes activated by the carrier components and the cytokines secreted by them play an important role in the immune response. (Guttormsen). There are different methods to detect an antigen specific T call mediated immune response. Tassignon J et al compared 4 different methods: ELISA, ELISPOT, intracytoplasmic detection and realtime RT-PCR to measure INF-gamma production of PBMCs before and after recall vaccination. In the present study we will use intracytoplasmatic detection of cytokines to define the cellular immune response.

The majority of publications, however, used serum antibody concentrations for defining protection. The pneumococcal IgG antibody assay used clinically for evaluating vaccine responses and the need for revaccination employs routineously the 23-valent polysaccharide vaccine as an antigen in the ELISA, thus measuring levels of IgG to all 23 serotypes included in the polysaccharide vaccine. The new pneumococcal serotype specific IgG assay as we will use in the present study, however, allows to determine the immune response to each single serotype and will provide important additional information regarding the protective status of an individual.

A recent consensus conference by the WHO recommends pneumococcal antibody concentrations of > 0.35 µg/ml as a surrogate for conjugate vaccine efficacy against invasive disease in general (<http://www.who.int/biologicals/Guidelines/vaccines.html>). This threshold level is only reliable after vaccination with the PCV7 where memory cells are induced. Thus, the estimation of seroprotection after vaccination with PPV23 is even more difficult: In contrast to PCV7, PPV23 does not prime the immune system for a rapid booster response. Therefore Sanders defined antibody titers of > 1.0 µg/ml for at least five of seven measured serotypes as predictive for a successful immunization after PPV23 vaccination. Since our patients did not respond to single polysaccharide vaccination and seemed to be at an increased risk of pneumococcal infections, we also chose antibody titers > 1.0 µg/ml as correlate of seroprotection.

**Aim of the study**

The aim of this study is to compare the quantitative antibody responses of PPV23 with those of a sequential schedule of PCV7 followed by PPV23 12 months later in adult renal transplant recipients by measuring the concentrations of serotype-specific IgG in sera using an isotype-specific ELISA that has recently been recommended by the World Health Organization.

For a subset of subjects the T-cell mediated immune response will be evaluated in both groups.

**Materials and Methods**

*Study population:*

Adult patients will be recruited at the transplant center of the Medical University of Vienna. It is estimated that 100-200 kidney transplantations take place per year.

Patients will be eligible if between 6 months and 3 years had passed since their kidney transplant and if they have stable allograft function, as evidenced by a creatinine level < 250 µmol/L for 1 month prior involvement.

Basic demographic data including age, gender, and medical history will be obtained.

Exclusion criteria included previous splenectomy, pneumococcal vaccination within the preceding 5 years, ongoing treatment for an episode of allograft rejection, any acute febrile illness in the 2 weeks prior to or at the time of enrolment, and use of intravenous immunoglobulin within preceding 6 month, have a history of severe allergic reactions or anaphylaxis. No changes to the current immunosuppression protocol will be made for the study.

Subjects will be exclude from participation in the evaluation of cellular immunity if they show a haemoglobin value < 10 g/dl.

*Study design and vaccines:*

A randomized, double blind, controlled trial will be performed. Eligible patients will be randomly assigned to receive a single dose of PCV7 (Prevenar; Wyeth Pharma GmbH) or PPV23 (Pneumo23, Sanofi-Pasteur-MSD, Lyon, France) in the deltoid muscle.

The PCV7 is a 7-valent pneumococcal conjugate vaccine containing 2µg polysaccharides of the serotypes 4, 14, 9V, 18C, 19F, 23F, respectively and 4 µg of the serotype 6B in 0.5 ml. The polysaccarides are conjugated to the carrier protein CRM197 and adsorbed to aluminum phosphate. PCV7 induces T cell dependent immune response that results in protective levels of antibody in infancy, as well as inducing booster response with subsequent doses.

The PPV23 is a polysaccharide vaccine containing 25 µg of 23 pneumococcal serotypes: 1–5, 6B, 7F, 8, 9N, 9V, 10A, 11A, 12F, 14, 15B, 17F, 18C, 19A, 19F, 22F, 23F, and 33F in 0.5 ml. This vaccine contains the individually extracted and purified capsular polysaccharides, which are combined into the final vaccine product. Polysaccharide vaccines are indicated for use in adults and children age > years who are at high risk for pneumococcal disease.

Participants will be enrolled into the study by the principal investigator. The vaccine will be assigned from a computer-generated randomization schedule, immediately before administration.

The administrator of the vaccine and the patient will be blinded to the type of vaccine given.

Patients randomized to group A will receive the PPV23 vaccine as early as 6 months after transplantation and the second dose of PPV23 12 months after the first vaccination.

Patients randomized to group B will receive the first PCV7 as early as 6 months after transplantation, followed by a PPV23 dose 12 months after the PCV7.

|  | 6 M after Tx | 18 M after Tx |
| --- | --- | --- |
| Regime 1 | PPV23 | PPV23 |
| Regime 2 | PCV7 | PPV23 |

*Study endpoints:*

Immunogenicity:

- Antibody concentrations after vaccination
- Fold increase of antibody response as compared to baseline 8 weeks after the vaccination
- Number of subjects with seroconversion defined as minimum 2-fold titer increase and a serum concentration of at least 1 µg/ml
- Number of subjects with at least > 2-fold increase for 2 of the 7 measured serotypes

For the subset of subjects included in the evaluation of cellular immunity

- T-cell response after each vaccination as determined by the frequency of cytokine producing T-cells induced by conjugated pneumococcal Ag
- Increase in frequency of cytokine producing T-cells induced by conjugated pneumococcal Ag after each vaccination as compared to baseline

*Safety data:*

Patients will be instructed to record local reactions /e.g. redness, swelling, and tenderness) and systemic reactions (e.g. fever, nausea, and vomiting).

At the time of vaccination, medical providers will be asked to record appearance of local reactions occurring within 30 minutes of injection. At the time of each vaccination visit patients will be given a diary card, thermometer and a measuring tape. On postvaccination days 1 through 3 patients will be asked to record presence or absence of redness and swelling (in millimetres), pain/tenderness (graded none, mild, moderate or severe), loss of appetite, vomiting, headache, fussiness, increased sleep or other potential adverse reactions. Data from the diary will be recorded as well as changes in medications or medical care.

Serum samples will be obtained immediately before, 8 weeks the first and the second vaccination.

In a subgroup of patients in each treatment regimen blood for cellular response will be taken immediately before vaccination and 7-10 days after vaccinations.

*Definitions and statistical methods:*

All patients will be included in the analysis as randomised.

Although the primary endpoint is the difference in antibody concentration as continuous variable between study groups we base our sample size calculation on the more conservative but clinically more useful binary variable vaccine response. A vaccine response by ELISA will be defined as a > 2 fold increase in prevaccination titer and an absolute postvaccination value of at least 1 µg/ml.

The study is powered to detect an improvement in vaccine response with the conjugate vaccine in any of the 7 serotypes. The study is set to yield a power of 80% at a significance level of 5% to detect a 35% improvement in the response rate for a given serotype. The response rate in the control group is expected at about 45%. Thus the calculated sample size will be 35 in each group. We added 10% to adjust for potential loss-to-follow-up which results in 39 patients in each group.

We expect antibodies being log-normally distributed. Geometric mean antibody concentrations (GMCs) for each pneumococcal serotype will be compared between the two groups by using the 2-tailed, unpaired Students’s t-test after log transformation of the raw data, given our distributional assumptions hold.

For binary variables we will calculate risk ratios and exact 95% confidence intervals. Vaccination efficacy (E_v_) will be calculated as: E_v_ = (1-RR) x 100. For hypothesis testing we will use the Fisher’s exact test.

All analysis will be conducted by using a 2-sided p value with a type I error rate of < 0.05.

*Determination of serum antibodies against vaccination antigens:*

Serum IgG antibodies against the seven pneumococcal conjugate polysaccharides included in the heptavalent vaccine were measured by enzyme-linked immunosorbent assays (ELISA). Serotype-specific IgG antibody concentrations to pneumococcal serotypes 4, 6B, 9V, 14, 18C, 19F and 23F were measured by reference ELISA according to the WHO protocol and to the publication of Wernette et al. [14, 15]. Briefly, 96-well microtiter plates (Greiner, Germany) were coated with previously determined optimal antigen concentrations (1 μg/ml for serotype 14; 2 µg/ml for serotypes 4, 9V, 18C and 5 μg/ml for serotypes 6B, 19F and 23F) and incubated at 37°C for 5 h in a humidified chamber. The coated plates were stored at 4°C and used within three weeks. Standard reference serum (89SF), kindly provided by Carl E. Frasch (Center for Biologics Evaluation and Research, U.S. Food and Drug Administration, Rockville, MD, USA), was used for assay standardization and was absorbed with 5 μg/ml of highly purified cell-wall polysaccharide (C-Ps) (Statens Serum Institute, Copenhagen, Denmark). Quality controls (QC) and serum test specimens were preincubated with 5 μg/ml C-Ps as well as pneumococcal serotype 22F capsular polysaccharide at a final concentration of 10 μg/ml to remove cross-reactive antibodies [16].

The coated plates were washed five times with 1 x Tris-buffered saline (TBS) containing 0.1% BRIJ35 (pH 7.2), then serially diluted 89SF, QC and serum samples were added in duplicate to the plates and incubated for 2 h at room temperature. Plates were washed again as described above and 50 μl of 1:5000 diluted goat anti-human IgG-alkaline phosphatase conjugate (Sigma) was added to each well. After incubation for 2 h at room temperature and a final washing step the plates were developed with 100 μl of nitrophenyl-phosphate substrate (Sigma) (1 mg/ml) for 30 min at room temperature. The enzyme reaction was stopped with 50 μl of 3M NaOH and the absorbance was measured at wavelength 405 nm in an ELISA reader. Concentrations of pneumococcal IgG antibody were calculated with a weighted log-logit computer program. The interassay coefficient of variation was about 20%.

### *Detection of antigen-specific T cell cytokine expression by flow cytometry:*

PBMC are isolated from heparinized blood by ficoll-diatrizoate centrifugation and plated out in 6-well plates at 5 x 10^6^/well. Cells are cultured in 5 ml ultra culture medium (UCM) (Bio Whittaker, Walkersville, Maryland) supplemented with L-glutamine (2 mM/l; Sigma, St. Louis, Missouri), gentamicin (170 mg/l; Sigma) and 2-mercaptoethanol (3.5 μl/l; Merck, Darmstadt, Germany) for 18 hours at 37° C in 5% CO_2_ and stimulated with or without pneumococcal antigen at a defined concentration. In order to amplify TCR signaling and to facilitate the initial phase of T cell activation the co-stimulatory MAb CD28 (Pharmingen, San Diego, California) is added at a final concentration of 5μg/ml to some wells. Brefeldin A (10μg/ml final concentration, Sigma) is added after 6 hours to block protein secretion. Cells are then harvested, fixed, and stained (MAb IFN-γ [clone: B27], fluorescein-isothiocyanate [FITC]-labeled; MAb IL-2 [MQ1-17H12], phycoerythrin [PE]-conjugated; IL-10 [JES3-9D7], PE-labeled; TNF-α [MAb11], PE-labeled, MAb TGF-β [TB21], PE-labeled), as reported previously. All MAb are purchased from Pharmingen except MAb TGF-β, which is purchased from IQ Products, The Netherlands. The Anti-CD4-MAb is allophycocyanin (APC)- and the anti-CD8-MAb is peridinin chlorophyll (PerCP)–labeled (Becton Dickinson, Mountain View, Calif.). At least 10^5^ cells are analyzed on a FACSCalibur (Becton Dickinson). Specificity of cytokine staining is confirmed by the absence of significant background in controls with isotype matched irrelevant MAbs. Data are analysed with CELLQuest software (Becton Dickinson), and results are expressed as the percentage of cytokine-producing cells in each CD4^+^ and CD8^+^ population.

*Detection of non-specific mitogen-induced T cell cytokine expression by flow cytometry:*

Flow cytometric assessment of intracellular cytokine expression is performed essentially according to the technique described previously. PBMC are isolated from heparinized blood and stimulated in UCM with phorbol 12-myristate 13-acetate (PMA, 10 ng/ml; Sigma) and ionomycin (1.25 μM; Sigma) in the presence of brefeldin A (1 μM; Sigma) for 4 hours at 37° C in 5% CO_2_. Cells are then harvested and fixed as described above. For the staining procedure the following monoclonal antibodies (MAbs) are used: cytokine-specific mouse anti-human MAb (IFN-γ [clone B27], fluorescein isothiocyanate [FITC]-conjugated); rat anti-human MAb (IL-2 [MQ1-17H12], IL-4 [MP4-25D2], IL-10 [JES3-9D7], IL-13 [JES10-5A2], TNF-α [Mab11], MAb TGF-β [TB21], all phycoerythrin [PE]-conjugated), the anti-CD4 MAb is allophycocyanin-, the anti-CD8 MAb is peridinin chlorophyll-conjugated; all cytokine-specific MAbs are purchased from Pharmingen (San Diego, California), except MAb TGF-β, which is purchased from IQ Products, The Netherlands. The surface marker-specific MAbs are purchased from Becton Dickinson (Mountain View, CA). All cytokine combinations are stained in conjunction with CD4 and CD8. Data are analysed with CELLQuest software (Becton Dickinson). Samples are gated on lymphocytes according to their light scatter characteristics and the results are expressed as the percentage of cytokine-producing cells in the CD4+ and CD8^+^ population, respectively.

**Schedule of events**

| **Procedure/assessment** | **Visit 1** | **Visit 1a**  **Day 7** | **Visit 2**  **Day 56** | **Visit 3**  **Day 365** | **Visit 3**  **Day 372** | **Visit 4**  **Day 421** |
| --- | --- | --- | --- | --- | --- | --- |
| Informed Consent | X |  |  |  |  |  |
| Inclusion/Exclusion Criteria | X |  |  |  |  |  |
| Medical History | X |  |  |  |  |  |
| Physical Examination | X |  |  |  |  |  |
| Blood Draw (Serology) | X |  | X | X |  | X |
| Blood Draw (Cellular Immunity) | X | X |  | X | X |  |
| Vaccination | X |  |  | X |  |  |
| Subject Diary (Part 1) | X |  | R |  |  |  |
| Subject Diary (Part 2) |  |  |  | X |  | R |
| AE Assessment | X | X | X |  | X | X |
|  |  |  |  |  |  |  |

## Time table and time schedule

Duration of the project: 2 years

*Year 1:*

Work package 1(WP1): Screening of patients

Inclusion/Exclusion

Informed Consent

Work package 2 (WP2): Randomization

Medical History

Blood drawn: red/white blood cell counts, pneumococcal antibodies, cellular immunity

Work package 3 (WP3): Determination of serotype specific pneumococcal antibodies

Work package 4 (WP4): Determination of cellular immunity

*Year 2:*

WP 3 and WP 4

BAR CHART:

|  | 1 | 2 | 3 | 4 | 5 | 6 | 7 | 8 | 9 | 10 | 11 | 12 | 13 | 14 | 15 | 16 | 17 | 18 | 19 | 20 | 21 | 22 | 23 | 24 |
| --- | --- | --- | --- | --- | --- | --- | --- | --- | --- | --- | --- | --- | --- | --- | --- | --- | --- | --- | --- | --- | --- | --- | --- | --- |
| WP1 | x | x | x | x | x | x |  |  |  |  |  |  |  |  |  |  |  |  |  |  |  |  |  |  |
| WP2 |  |  | x | x | x | x | x | x | x | x | x | x | x | x | x | x | x | x | x | x | x | x | x | x |
| WP3 |  |  | x | x | x | x | x | x | x | x | x | x | x | x | x | x | x | x | x | x | x | x | x | x |
| WP4 |  |  | x | x | x | x | x | x | x | x | x | x | x | x | x | x | x | x | x | x | x | x | x | x |

# **References**

Ahmed F, Steinhoff MC, Rodriguez-Barradas MC, et al. Effect of human immunodeficiency virus type 1 infection on the antibody response to a glycoprotein conjugate pneumococcal vaccine: results from a randomized trial. J Infect Dis. 1996; 173: 83-90

Avetisyan G, Ragnavölgyi, Toth GT, et al. Cell-mediated immune responses to influenza vaccination in healthy volunteers and allogeneic stem cell transplant recipients. Bone Marrow Transplantation 36; 411-415.

Butler JC, Breiman RF, Campbell JF, et al. Pneumococcal polysaccharide vaccine efficacy. An evaluation of current recommendations. JAMA 1993; 270: 1826-31.

Centers for Disease Control and Prevention. Prevention of pneumococcal disease: recommendations of the Advisory Committee on Immunization Practices (ACIP). MMWR Morb Mortal Wkly Rep 1997;46: 1-24.

Chan CY, Molrine DC, George S et al. Pneumococcal conjugate vaccine primes for antibody responses to polysaccharide pneumococcal vaccine after treatment of Hodgkin’s disease. J Infect Dis 1996; 173:256-8.

Eskola J, Antilla M. Pneumococcal conjugate vaccines. Pediatr Infect Dis J 1999; 18:543-551.

Eskola J. Immunogenicity of pneumococcal conjugate vaccines. Pediatr Infect Dis J 2000;19:388-93

Eigenberger K, Sillaber Ch, Greitbauer M, et al. Antibody response to pneumococcal and hemophilus vaccinations in splenectomized patients with hematological malignancies or trauma. Wien Klin Wochenschr 2007; 119: 228-234

Fedson DS, Musher DM, Eskola J. Pneumococcal vaccine. In: Plotkin SA, Orenstein WA, eds. Vaccines. 3^rd^ ed. Philadelphia: WB Saunders, 1998.

Feikin DR, Elie CM, Goetz MB, et al. Randomized trial of quantitative and functional antibody responses to a 7-valent pneumococcal vaccine ans/or 23-valent polysaccharide vaccine among HIV-infected adults. Vaccine 2001;20:545-53

Gaillat J, Zmirou D, Mallaret MR, et al. Clinical trial of an antipneumococcal vaccine in elderly subjects living in institutions. Rev Epidemiol Sante Publique 1985; 33: 437-44.

Guttormsen HK, Sharpe A, Chandraker A, et al. Cognate stimulatory B-cell T-cell interaction are critical for T-cell help recruited by glycoconjugate vaccines. Infect Immun 1999; 67: 6375-84.

Jackson LA, Neuzil KM, Yu O, et al. Effectiveness of pneumococcal polysaccharide vaccine in older adults. N Engl J Med 2003; 348:1747-55.

Lindemann M, Witzke O, Tütkes P et al. ELIspot assay as a sensitive tool to detect cellular immunity following influenza vaccination in kidney transplant recipients. Clin Immunol 2006; 120: 342-348.

Ling Li P. Michaels MG, Green M et al. Safety and Immunogenicity of the American Academy of Pediatrics-recommended sequential pneumococcal conjugate and polysaccharide vaccine schedule in pediatric solid organ transplant recipients. Pediatrics 2005; 116: 160-167.

Linnemann CC Jr, First MR. Risk of pneumococcal infections in renal transplant patients. JAMA 1979; 241:2619-21.

Linnemann CC, First MR, Schiffman G. Revaccination of renal transplant and hemodialysis recipients with pneumococcal vaccine. Arch Intern Med 1986; 146:1554-6

McElhaney JE, Xie D, Hager WD, et al. T cell responses are better correlates of vaccine protection in the elderly. The Journal of Immunology 2006; 176: 6333-633

Nichol KL. The additative benefits of influenza and pneumococcal vaccinations during influenza seasons among elderly persons with chronic lung disease. Vaccine 1999; 17 suppl 1: S91-3.

Ortqvist A, Hedlund J, Burman LA et al. Rdomised trial of 23-valent pneumococcal capsular polysaccharide vaccine in prevention of pneumonia in middle-aged and elderly people. Swedish Pneumococcal Vaccination Study Group. Lancet 1998; 351: 399-403.

Powers DC, Anderso EL, Lottenbach K, et al. Reactogenicity and immunogenicity of a protein-conjugated pneumococcal oligosaccharide vaccine in older adults. J Infect Dis 1996; 173:1014-1018

Ramharter M, Winkler H, Kremsner PG, et al. Age-dependency of Plasmodium falciparum specific and non-specific T cell cytokine responses in individuals from a malaria-endemic area. Eur Cytokine Netw 2005; 16: 135-43.

Rendi-Wagner P, Georgopoulos A, Kundi M, et al. Prospective surveillance of incidence, serotypes and antimicrobial susceptibility of invasive S. pneumoniae among hospitalized children in Austria. JAC 2004; 53: 826-831.

Sanders LA, Rijkers GT, Kuis W, et al. Defective anti-pneumococcal polysaccharide antibody response in children with recurrent respiratory tract infections J Allergy Clin Immunol 1993; 91: 110-119.

Shelly MA, Jacoby H, Riley B, et al. Comparison of pneumococcal polysaccharide and CRM197-conjugated pneumococcal oligosaccharide vaccines in young and elderly adults. Infect Immun 1997; 65: 242-247

Rose MA, Schubert R, Strnad N et al. Priming of Immunological Memory by pneumococcal conjugate vaccine in children unresponsive to 23-valent polysaccharide pneumococcal vaccine. Clin Diagn Lab Immunol 2005; 12; 1216-1222.

Shrimpton A, Duddrige M, Ziegler-Heitbrock L. Vaccination with polysaccharide-conjugate vaccines in adult patients with specific antibody deficiency. Vaccine 2006; 24: 3574-80.

Vernacchio L, Romero-Steiner S, Martinez JE, et al. C of an opsonophagocytic assay and IgG ELISA to assess responses to pneumococcal polysaccharide and pneumococcal conjugate vaccines in children and young adults with sickle cell disese J Infect Dis 2000; 181:1162-6.

Stein KE. Thymus independent and thymus dependent responses to polysaccharide antigens J Infect Dis 1992; 165 (suppl):S49-52.

Stoehr GA, Rose MA, Eber SW, et al. Immunogenicity of sequential pneumococcal vaccination in subjects splenectomised for hereditary spherocytosis. BJH 2005; 132: 788-90.

Winkler S, Necek M, Winkler H, et al. Increased specific T cell cytokine responses in patients with active pulmonary tuberculosis from Central Africa. Microbes and Infection 2005; 7: 1161-1169.
